# Comprehensive assessment of 18AA amino acid injections based on Chinese guidelines: A narrative review

**DOI:** 10.1097/MD.0000000000045948

**Published:** 2025-11-21

**Authors:** Jiabao Li, Xiao Hu, Siyong Huang, Jisheng Chen

**Affiliations:** a Key Specialty of Clinical Pharmacy, The First Affiliated Hospital of Guangdong Pharmaceutical University, Guangzhou, Guangdong, China.

**Keywords:** 18AA, compounded amino acid injection, drug evaluation, health technology assessment

## Abstract

By systematically evaluating nine 18AA compounded amino acid injections using a percentage scoring method, we aim to provide a basis for hospitals to select compounded amino acid injections. According to the Rapid Guide for Drug Evaluation and Selection in Chinese Medical Institutions (Second Edition) released in 2023, relevant databases such as PubMed, Cochrane, Embase, drug labels, and clinical guidelines were searched for drug information. We used a percentage scoring method to score the nine 18AA compound amino acid injections marketed in China on 5 dimensions, including pharmacological properties (28 points), efficacy (27 points), safety (25 points), economy (10 points), and other attributes (10 points). We classified them into recommendation levels based on the scoring results, to objectively select and evaluate medicines. The final assessment result scores from highest to lowest were 18AA (71.50 points), 18AA-VII (66.77 points), 18AA-IX (66.63 points), 18AA-I (63.39 points), 18AA-II (61.56 points), 18AA-III (61.23 points), 18AA-V-SF (61.12 points), 18AA-V (58.75 points), and 18AA-IV (55.29 points). In the comprehensive overall assessment of amino acid formulations, 18AA-IX and 18AA-VII scored the highest in pharmacological properties and effectiveness, 18AA-IX and 18AA-V-SF excelled in safety, and 18AA scored the highest in economics and other attributes. When a healthcare organization introduces 18AA compounded amino acid injections to their hospital, they can refer to the assessment results and use the top 3 recommended medications: 18 AA, 18 AA-VII, and 18 AA-IX. Meanwhile, 18AA, which has the highest evaluation score and is >70, should be highly recommended as a new drug introduction and prioritized during drug adjustment and retention.

## 1. Introduction

Amino acids, the fundamental components of proteins essential for the human body, have a significant role in human metabolism and nutritional therapy.^[1]^ 18AA compound amino acid injection is a crucial nitrogen source for parenteral nutrition. It can provide a balanced composition of amino acids to meet the nitrogen demand of patients, ensuring the safety and efficacy of their nutritional therapy. There are 9 kinds of 18AA compound amino acid injections marketed in China, including 18 AA, 18 AA-I, 18 AA-II, 18 AA-III, 18 AA-IV, 18 AA-V, 18 AA-V-SF, 18 AA-VII, and 18 AA-IX. The nomenclature of these products usually involves the number of amino acids used in conjunction with the number of different dosing regimens or their English abbreviation with a suffix. Furthermore, 18 AA is a crucial amino acid in parenteral nutrition. Amino acids are divided into numerous proportional schemes based on different concentrations, nitrogen contents, and amino acid species, such as 18AA-V-SF, which is said to contain 18 kinds of amino acids. The 5th proportioning scheme is configured as a compound liquid, and SF means no sulfite antioxidants. And 9 kinds of 18aa compound amino acid injection can be categorized into balanced type and disease-applicable type according to their function and use. In addition to 8 kinds of essential amino acid (EAA), the balanced amino acids also contain 8 to 12 kinds of nonessential amino acid (NEAA), which are formulated in the ratio of 1:1 to 1:3, and are mainly used for patients with insufficient protein intake, absorption obstacles, or nutritional inability to meet the needs of the clinic to provide essential nutritional support for such patients. Disease-applicable amino acids are divided into liver disease-applicable, kidney disease-applicable, and trauma-applicable according to the amino acid metabolism characteristics of different diseases in consideration of the more appropriate types of amino acids to be supplemented to patients in different disease states, among which 18AA-IX belongs to kidney disease-applicable, 18AA-VII belongs to trauma-applicable, and the others belong to a balanced type. Parenteral nutrition is essential for patients who cannot meet their nutritional needs through the gastrointestinal tract due to conditions such as severe digestive diseases, postoperative recovery, critical illnesses, malignancies, and chronic malnutrition. These conditions necessitate alternative nutritional support to maintain metabolic functions and improve patient outcomes. Due to the wide variety of marketed 18AA compounded amino acid injections and the lack of systematic evaluation of drug dissimilarities, the National Health Commission 2023 released the Second National Catalog of Priority Drugs for Monitoring and Rational Use,^[2]^ in which compounded amino acids were included, in an attempt to further strengthen the rational use of clinical medication management. This study was based on the “Rapid Guide for Drug Evaluation and Selection in Chinese Medical Institutions (Second Edition)” (hereinafter referred to as the “Second Edition”),^[3]^ which systematically evaluated 18AA compound amino acid injections in 5 aspects, namely, pharmacological properties, efficacy, safety, economy, and other attributes, to gain a comprehensive understanding of their characteristics and application effects, to optimize the structure of drug varieties in hospitals and the drug catalog, and to provide a better solution for the healthcare institutions in terms of It can provide a reference for medical institutions in drug selection and clinical decision-making, especially for other countries with low and middle incomes, low medical standards or medical institutions that cannot carry out drug evaluation on their own.

## 2. Information and methodology

### 2.1. Evaluation of medicines

Because multiple manufacturing units exist for the nine 18 AA compounded amino acid injections, the selection of drugs to be evaluated was made in the following order.

Listed in China; original research drug; passed the consistency evaluation; the 1st place of the selected drug in the national collection; the drug that has won a large number of bids in the nationwide provincial centralized banded purchasing of medicines; and the production unit is in the list of the top 100 pharmaceutical industry of the Ministry of Industry and Information Technology or among the world’s top 50 pharmaceutical companies in terms of sales volume. The final medicines included in the evaluation are shown in Table 1.

**Table 1:** Medicines included in the evaluation.

	18AA	18AA-I	18AA-II	18AA-III	18AA-IV	18AA-V	18AA-V-SF	18AA-VII	18AA-IX
Production unit	Guangzhou Green Cross Pharmaceutical Co.	Cisen Pharmaceutical Co., Ltd.	Guangdong Litai Pharmaceutical Co.	Guangdong Litai Pharmaceutical Co.	Guangdong Litai Pharmaceutical Co.	Fuzhou Fu Yao Pharmaceutical Co., Ltd.	Hubei Yidian Pharmaceutical Co.	Liaoning Haisco Pharmaceutical Co., Ltd.	Liaoning Haisco Pharmaceutical Co., Ltd.
Dosage forms	250 mL:12.5 g	250 mL:17.5 g	250 mL:21.25 g	250 mL:25.90 g	250 mL:8.70 g	250 mL:8.06 g	250 mL:8.06 g	200 mL:20.65 g	200 mL:12.25 g
Eligible for selection	①, ⑤	①, ⑤, ⑥	①, ⑤	①, ⑤	①, ⑤	①, ⑤	①, ⑤	①, ③	①, ③

### 2.2. Evaluation basis

This study is based on “The Second Edition,” which adopts the mini-health technology assessment combined with the system of objectified judgment analysis. The evaluation dimensions and weights were determined by the guideline guidance group and the expert group through the Delphi method.^[3]^ In addition, compared with the 1st edition of this guideline, “The Second Edition” has revised and refined the evaluation indicators, so that the quantitative scoring can better reflect the priority of drugs in medical institutions, and the scoring items are more detailed, clear, and easy to operate. “The Second Edition” is a set of quantitative scoring systems for drug evaluation and selection in Chinese medical institutions, which is widely used in China.

The evaluation included 5 aspects: pharmacological properties (28 points), efficacy (27 points), safety (25 points), economy (10 points), and other attributes (10 points). In terms of pharmacological properties, it mainly evaluates the drug’s pharmacological effects, in vivo processes, whether the pharmacology and methods of use are clear, the length of the drug’s validity period, and storage requirements. In terms of drug effectiveness, it mainly evaluates the clinical efficacy of the drug and the degree of recommendation of relevant authoritative professional information such as clinical guidelines or expert consensus. In terms of safety, it mainly evaluates adverse events, the use of drugs in special populations, drug interactions, etc. In terms of economy, the average daily therapeutic cost of the drug was evaluated. In addition, the supply information of the drug is also evaluated.

### 2.3. Detailed criteria for quantitative assessment of drug selection in hospitals

The specific index system and weighting coefficients were as follows: pharmaceutical properties included pharmacological effects (5 points), in vivo processes (5 points), pharmacy and methods of use (12 points), storage conditions (4 points), and expiry date (2 points), totaling 28 points; efficacy included indications (5 points), guideline recommendations (12 points), and clinical efficacy (10 points), totaling 27 points; safety includes adverse reactions (8 points), special populations (11 points), adverse reactions due to drug interactions (3 points), and others (3 points), totaling 25 points; economy includes drugs with the same generic name (3 points) and alternative drugs for the main indications (7 points), totaling 10 points; and other attributes include the National Health Insurance (NHI; 3 points), the national essential medicines (3 points), the national centralized purchasing of medicines (1 point), the original research drug/reference listed drug/consistency evaluation (1 point), the status of the manufacturing company (1 point), and the global use of the drug (1 point), totaling 10 points.

### 2.4. Data source

The sources of information include drug manuals, drug registration information, DrugWise.com DrugWise data on drug winning prices (https://db.yaozh.com/), the NHI Information Database (https://code.nhsa.gov.cn), the Drug Evaluation Centre of the State Food and Drug Administration (FDA; https://www.cde.org.cn) and other authoritative medical institutions information query platform. English databases PubMed, Embase, Update databases, and Cochrane Library, as well as Chinese databases Chinese Biomedical Sciences, China Knowledge Network, Wanfang Database, Medication Assistant and Medical Pulse Database (https://www.medlive.cn), to search for 5 years’ worth of information published by authoritative institutions within 5 years.

### 2.5. Analysis and evaluation

Drugs included in the assessment were scored according to the drug assessment guidelines, based on evidence gathered by searching relevant databases. If scoring breakdown rules were not available, experts in the field were invited to refine them. Evaluations were conducted independently by 2 clinical pharmacists. If there were conflicting results, experts in the relevant field were invited to discuss the evaluation results. The results of the evaluation are ultimately used for decision-making on drug selection and clinical medication planning in healthcare organizations.

## 3. Results

### 3.1. Pharmacological properties

The pharmacological assessment was guided by the 2nd edition and was based on drug inserts, registration data, and relevant research literature, as well as the following databases: PubMed, Embase, China Knowledge Network, and Wanfang database. The objective was to compare the differences in details and confirm whether the drugs were substitutable with each other by scoring the selected drugs in 5 aspects, pharmacological effect, in vivo processes, pharmacy and the use of drugs, storage conditions, and expiration date of the drugs.

In terms of pharmacological effects, 9 drugs scored 4 points for their precise clinical efficacy and clear mechanism of action; in terms of in vivo processes, 9 drugs scored 3 points for their clear in vivo processes and incomplete pharmacokinetic parameters. In terms of pharmacology and method of use, the main components and excipients of the 9 drugs were clear, all scored 2 points; specifications and packaging were suitable for clinical application/dose adjustment, all scored 2 points; dosage form was intravenous drip injection, all scored 1 point; the dosage of the drug administration is needed to calculate the doses according to the mass or body surface area, all scored 1 point; the frequency of drug administration was ≤1 time/day, all scored 2 points, and the use of the drug needed medical staff to the frequency of administration is ≤1 time/day, all scored 2 points; the use of medical personnel is needed to administer the drug, the convenience of use are scored 1 point. In terms of storage conditions, 18AA-III, 18AA-IV, 18AA-V, and 18AA-V-SF are stored in a cool and dark place (no more than 20°C), scored 2 points, and the other medicines are stored at room temperature, of which 18AA-I and 18AA-II need to be protected from light/shade, scored 3 points, and 18AA, 18AA-VII, and 18AA-IX do not need to be protected from light/shade, scored 3 points and avoid light/shade, scored 4 points. Regarding the validity period of the drugs, 18AA is valid for 18 months and scored 0.5 points, while the validity period of the rest of the drugs is 24 months and scored 1 point. Table 2 displays the results of the pharmacologic properties evaluations for the nine 18AA compounded amino acid injections.

**Table 2:** Pharmacological properties score results.

Pharmacological properties (28 points)	Grade	18AA	18AA-I	18AA-II	18AA-III	18AA-IV	18AA-V	18AA-V-SF	18AA-VII	18AA-IX
*Pharmacological effect (5 point*)										
Clinical efficacy obvious, action mechanism is clear, mechanism or target is innovative	5									
Clinical efficacy obvious, mechanism is clear	4	4	4	4	4	4	4	4	4	4
Clinical efficacy general, mechanism is unclear	2									
Clinical efficacy poor, mechanism is unclear	1									
*In vivo processes (5 points*)										
In vivo process clear, pharmacokinetic parameters complete	5									
In vivo process clear, pharmacokinetic parameters not complete	3	3	3	3	3	3	3	3	3	3
In vivo process not clear, pharmacokinetic parameters not complete	1									
*Pharmacy and the use of drugs (12 point*)										
Main ingredients and excipients	2	2	2	2	2	2	2	2	2	2
Specification and packaging	2	2	2	2	2	2	2	2	2	2
Dosage form (oral/inhalation/topical preparations (2 pts), subcutaneous/intramuscular injections (1.5 pts), intravenous drip/injection (1 pt))	2	1	1	1	1	1	1	1	1	1
Dose of administered (fixed dosage (2 pts), dosage adjustment required during use (1.5 pts), dosage based on body mass or body surface area (1 pt))	2	1	1	1	1	1	1	1	1	1
Administration frequency (1x/d, (2 pts); 2x/d (1.5 point); ≥3x/d (1 pts))	2	2	2	2	2	2	2	2	2	2
Convenient use (self-administered without assistance (2 pts), self-administered without assistance, with help or training (1.5 pts), administered by medical personnel (1 pt))	2	1	1	1	1	1	1	1	1	1
*Storage conditions (4 points*)										
Room temperature storage	3	3	3	3					3	3
Cool storage	2				2	2	2	2		
Refrigerated/frozen storage	1									
No need for shading/sheltering	1	1							1	1
*Expiration dates (2 point*)										
>60 mo	2									
≥36 mo, <60 mo	1.5									
≥24 mo, <36 mo	1		1	1	1	1	1	1	1	1
≥12 person-mo, <24 mo	0.5	0.5								
0.25 < 12 mo	0.25									
The results of the pharmacological properties	28	20.5	20	20	19	19	19	19	21	21

### 3.2. Effectiveness

#### 3.2.1. Indications

Checking the relevant drug specifications and literature, 9 drugs are mainly used for the treatment of malnutrition, hypoproteinaemia, and amino acid supplementation before and after surgery and cannot be orally supplemented with amino acids. In the period of renal failure, the ratio of amino acid and protein in the body is imbalanced, the total amount of EAA, histidine, and lysine content decreases significantly, the nitrogen content in the body rises, and the body uses nitrogen to synthesize a large amount of NEAA, which causes an imbalance in the ratio of EAA/NEAA, leading to the deterioration of the condition, resulting in the body’s negative nitrogen balance and the rise of blood urea nitrogen, so the patients with renal failure should be treated with a higher EAA/NEAA ratio^[4]^ or compound amino acid injection of EAA and histidine.^[5]^ Among the domestically marketed compound amino acid injections, 9AA has the highest EAA/NEAA ratio of 20.54, followed by 18AA-IX with 3.30. Therefore, 18AA-IX can be considered the 2nd choice of clinical drugs for patients with indications and comorbid nephropathy, with a score of 3 points. Due to the rapid increase in protein catabolism in patients with severe stress, such as extensive burns, major surgery, and severe trauma, it is easy to have a negative nitrogen balance to make the condition worse and amino acid injections with higher calorie distribution density and branched-chain amino acid content should be given to provide the body with enough nitrogen sources needed for protein synthesis, enhance resistance and promote wound healing.^[4,6]^ The compound amino acid injection that meets this requirement includes 15-HBC and 18AA-VII. 15-HBC contains up to 45% branched-chain amino acids, whereas 18AA-VII contains fewer branched-chain amino acids than 15-HBC, but it increases the EAA content as well as the type of NEAA, and the total amino acid content is up to 10.3%. Therefore, 18AA-VII can be considered a clinical 2nd-choice drug for patients who meet the indications and have extensive trauma, scoring 3 points. The remaining 7 drugs had more clinically available drugs in terms of indications, and all of them scored 1 point.

#### 3.2.2. Guideline recommendations^[7,8,9,10,11]^

Checking the guideline query websites, such as the Medical Pulse, Medication Assistant, Drugwise, and Update databases, 9 drugs were recommended by expert consensus. In addition, 18AA-V-SF was recommended by guideline level II and below other evidence, so this item of 18AA-V-SF was scored as 7, and the other 8 drugs were scored as 5. The guideline recommendations are shown in Table 3.

**Table 3:** Recommendations in domestic and foreign guides and consensus.

Guide name	Guideline makers and sources	Recommended medications	Recommended content
Chinese Expert Consensus on Supplemental Parenteral Nutrition for Adults^[7]^	CSPEN	Balanced amino acids (18AA, 18AA-I, 18AA-II, 18AA-III, 18AA-IV, and 18AA-V)	Amino acid solution is the form of protein supply in PN formulas, and better nutritional support can be achieved by choosing the ideal formulation of amino acid solution. Generally, balanced amino acid solutions can meet the nitrogen requirements of most critically ill patients.
Expert consensus on the clinical application of compound amino acid injection^[8]^	CSPEN	Compound Amino Acids Injection (18AA, 18AA-I, 18AA-II, 18AA-III, 18AA-IV, 18AA-V, 18AA-V-SF, 18AA-VII, and 18AA-IX)	Recommendation 10: Give priority to balanced compound amino acid injection for general patients, and give priority to disease-applicable compound amino acid injection such as liver disease-applicable, kidney disease-applicable and trauma-applicable compound amino acid injection for special diseases, such as chronic liver disease, chronic kidney disease, and trauma-applicable compound amino acid injection.
Chinese Expert Consensus on Nutritional and Exercise Interventions for Muscle Weakening Syndrome (Abridged)^[9]^	Geriatric Nutrition Branch of the Chinese Nutrition Society, Clinical Nutrition Branch of the Chinese Nutrition Society, Geriatric Nutrition Support Group of CSPEN	Essential amino acid	Resistance-based exercises (e.g., seated leg raises, static squats against a wall, dumbbell lifting, elastic band pulling, etc) are effective in improving muscle strength and body function; supplementation with essential amino acids or high-quality proteins is even more effective. (A)
Guideline for clinical application of parenteral and enteral nutrition in adults patients in China (2023 edition)^[10]^	CSPEN	Sulphite-free antioxidant compounded amino acid formulation (18AA-V-SF)	Amino acids, as a source of nitrogen for the body, are recommended to be supplied in adequate amounts of nonprotein calories to avoid wastage; the use of sulfite-free antioxidant compounded amino acid preparations is recommended to minimize liver damage.
Chinese Expert Consensus on Adult Home Parenteral Nutrition^[11]^	CSPEN	Balanced amino acids (18AA, 18AA-I, 18AA-II, 18AA-III, 18AA-IV, and 18AA-V)	Compounded amino acid solutions are the main form of protein supply in HPN formulations. It is currently believed that balanced amino acid solutions can satisfy the nitrogen requirements of most patients and can achieve better nutritional supportive treatment results

#### 3.2.3. Clinical efficacy

RCTs (Randomized Controlled Trial) and meta-analyses were reviewed to determine clinical outcomes. The primary outcome endpoints were overall mortality^[12]^; secondary outcome endpoints were the length of hospital stay and changes in inflammatory markers (e.g., C-reactive protein, calcitonin, etc), and nutritional markers (e.g., albumin, total protein, and nitrogen balance).^[12,13]^

In terms of primary efficacy, a growing number of studies have shown that the amount of protein supplied is an independent factor in the efficacy of clinical nutritional support and clinical outcomes.^[7]^ In critically ill and malnourished patients, the higher the protein intake is, the lower the morbidity and mortality.^[12]^ Therefore, a higher concentration of amino acids in balanced compound amino acid injections may improve survival and disease remission rates, thus improving primary efficacy endpoints.

In terms of secondary efficacy, studies have reported that amino acid metabolism has a profound effect on cellular function and that immune cells are dynamically activated in response to infection and changes in the organizational environment, suggesting that they are heavily dependent on metabolic status.^[14]^ At the same time, evidence suggests that some amino acids can modulate the immune response through multiple biochemical pathways, reducing inflammatory responses and avoiding complications.^[13,15,16]^ Therefore, elevating the amino acid concentration within a balanced compound amino acid injection could enhance amino acid metabolism, stimulate the immune response, ameliorate inflammatory markers, and ultimately heighten secondary efficacy endpoints.

Given that both primary and secondary efficacy endpoints are tied to amino acid concentration, they are separated into 3 tiers, and subsequently, primary and secondary efficacy endpoints are graded based on varying concentration levels. Amino acid concentrations were classified into 3 categories: <5%, 5%–10%, and > 10%. Scores of 4, 5, and 6 were assigned for the primary efficacy endpoints, and 2, 3, and 4 were assigned for the secondary efficacy endpoints. The amino acid concentrations of the 9 drugs are shown in Table 4.

**Table 4:** Amino acid concentrations of the 9 drugs.

Drug name	18AA	18AA-I	18AA-II	18AA-III	18AA-IV	18AA-V	18AA-V-SF	18AA-VII	18AA-IX
Amino acid concentration %	5	7	8.50	10.36	3.48	3.22	3.22	10.33	6.13

For clinical efficacy, 18AA-IV, 18AA-V, and 18AA-V-SF scored a total of 6 points; 18AA, 18AA-I, and 18AA-II scored a total of 8 points; and 18AA-III scored a total of 10 points. According to the expert consensus,^[8]^ 18AA-VII and 18AA-IX are trauma-applicable and nephropathy-applicable compounded amino acid injections, respectively, and the amino acid content ratios are in line with the medication needs of the corresponding patient populations; therefore, 18AA-VII and 18AA-IX can be scored with a total of 6 points for primary efficacy endpoints and 4 points for secondary efficacy endpoints, for a total of 10 points. The efficacy scores results are shown in Table 5.

**Table 5:** Efficacy score results.

Efficacy (27 points)	Grade		18AA	18AA-I	18AA-II	18AA-III	18AA-IV	18AA-V	18AA-V-SF	18AA-VII	18AA-IX
*Indications (5 points*)											
Clinically necessary and preferred		5									
Clinical need, second choice		3								3	3
More drugs available		1	1	1	1	1	1	1	1		
*Guideline recommendations (12 points*)											
Diagnostic and treatment protocols/clinical pathways, consensus/management approaches issued by national health administrative agencies, etc, and guideline Level I recommendations (12 points for Level A evidence, 11 points for Level B evidence, and 10 points for Level C evidence and others).		12									
Guideline Level II and below recommendations (9 points for Level A evidence, 8 points for Level B evidence, and 7 points for Level C evidence and others).		9							7		
Expert Consensus Recommendations (6 points for consensus issued by a Society organization based on systematic evaluation, 5 points for consensus issued by a Society organization, and 4 points for others).		6	5	5	5	5	5	5		5	5
Systematic Evaluation/Meta-Analysis (3 points for large sample, high quality systematic evaluation/meta-analysis, 2 points for small sample, low quality systematic evaluation/meta-analysis, and 1 point for systematic evaluation/meta-analysis of non-RCT studies).		3									
*Clinical efficacy (10 points*)											
Primary efficacy endpoints		6	5	5	5	6	4	4	4	6	6
Secondary efficacy endpoints		4	3	3	3	4	2	2	2	4	4
The results of the efficacy		27	14	14	14	16	12	12	14	18	18

RCT, Randomized Controlled Trial.

### 3.3. Safety

The safety assessment of the drugs to be selected was based on 4 aspects, namely, adverse drug reactions (ADR), drug use in special populations, drug interactions, and others, based on the drug inserts, drug registration information, information released on government websites such as the FDA and National Medical Products Administration (NMPA), and relevant Chinese and English literature.

#### 3.3.1. Adverse reactions

Refer to CTCAE-V5.0.^[17]^ Grading to determine the severity of adverse reactions caused by the selected medications. Compound amino acid injection of 18AA and similar preparations may result in adverse reactions such as fever, headache, nausea, vomiting, palpitations, and allergic reactions. Zhang et al^[18]^ concluded that compound amino acid injection (18AA) had allergic reactions and phlebitis as its main ADR, and in severe cases, anaphylaxis, when observing its effect on fetal intrauterine growth restriction treatment. Chen Huixia et al^[19]^ examined 45 patients who experienced adverse reactions to compound amino acid injection (18AA) between 2014 and 2017. The results showed that 80.00% of the total adverse reactions occurred on the day of injection, with a lower incidence of adverse reactions as time elapsed.^[20–22]^ Combined with the CTCAE-5.0 grading, most adverse reactions associated with the compound amino acid injection were mild, and they typically resolved spontaneously without treatment. However, moderate adverse reactions were scored as 3 when using the grading system endorsed by the US FDA.^[23]^ In a study monitoring 476 cases of adverse reactions to the injection, 86 cases of allergy were reported, with 21.1% of the adverse reactions occurring in products containing sulfites and 19.7% in products without sulfites. Consequently, the study concluded that allergic reactions should be considered serious adverse events associated with compound amino acid injection. Due to insufficient data on adverse reactions, it was not feasible to calculate reaction incidence scores. It is notable that compound amino acid injections in China commonly contain antioxidants, such as sulfites, to enhance preparation stability. However, sulfites pose a risk of inducing hypersensitivity reactions and damaging tissues and organs.^[21,24]^ To increase the risk of allergic reactions to compound amino acid injections and increase the incidence of serious adverse events, the sulfite-free preparation 18AA-V-SF scored 5 points for serious adverse reactions, and the rest scored 4 points.

#### 3.3.2. Special populations

##### 3.3.2.1. Children

The dosage and precautions for infants and young children are mentioned in the instruction manuals of 18AA, 18AA-I, 18AA-II, 18AA-V, and 18AA-V-SF, scoring 2 points; the instruction manuals of 18AA-VII and 18AA-IX mention that it is available for children over 3 months old, scoring 1.9 points, and the rest of the points are not scored.

##### 3.3.2.2. Elderly

Zhang Qian^[25]^ assessed the use of amino acid injection in the hospital from January to July 2018 and found that the average age of 308 patients was 64.6 years old, of which 288 patients used 18AA-II, accounting for 93.51%; Qin Shouquan^[26]^ observed the use of in-hospital total parenteral nutrition and found that of the 246 patients, 152 patients were aged ≥ 60 years, accounting for 61.78%, and 64 patients, accounting for 26.02%, used 18AA-II or 18AA-II-SF. Therefore, it was presumed that 18AA-II was available for elderly patients. This item scored 1 point for the 18AA-II and 1 point for the rest of the similar preparations whose instructions mentioned use in elderly individuals.

##### 3.3.2.3. Women during pregnancy

NEAAs and EAAs regulate key metabolic pathways in the body during pregnancy and play an important role in regulating several aspects of gene expression, immunity, and neurological function to maintain fetal growth and development.^[27]^ It has been suggested that the uterus, placenta, and maternal physical condition are important influences on fetal growth and development and that balanced energy/protein supplementation may improve fetal growth and reduce the risk of fetal and neonatal mortality.^[18]^ In contrast, in early pregnancy, maternal plasma concentrations of NEAAs and EAAs decrease and remain low. Therefore, adequate amino acid supplementation is needed in early pregnancy. Liqiong Ma^[28]^ found that early administration of compound amino acid injection could treat fetal growth restriction in pregnant women with an overall effective rate of 67.79%, which proved that compound amino acid injection could be used in pregnant women with therapeutic effects. However, considering that except for 18AA-VII and 18AA-IX, which are mentioned in the instruction manual that they should be administered to pregnant patients only when the benefits of treatment outweigh the dangers, the rest of the drug instruction manuals do not mention it, and the expert consensus on the clinical application of compound amino acid injection issued in 2019^[8]^ does not recommend the use of compounded amino acids in pregnancy and labor. Therefore, on balance, it was decided to give discretionary scores, with all 9 medicines receiving a score of 0.5.

##### 3.3.2.4. Lactating women

Animal studies have demonstrated that providing additional amino acids, including serine and glutamine, can enhance the growth and metabolism of lactating animals.^[29]^ Furthermore, administering intravenous amino acid complexes can considerably enhance the well-being of postnatal animals.^[30]^ Therefore, during lactation, it is feasible to supplement with appropriate amino acids. For the human body, the World Health Organization recommends exclusive breastfeeding for 6 months. The mother’s physical condition and nutritional intake are prerequisites for milk production. If the mother’s nutrition is inadequate, it may result in decreased milk production and potentially affect her health.^[31]^ However, as lactation continues, the content of total amino acids and each amino acid in breast milk decreases. Free and conjugated amino acids play distinct physiological roles during early life. Infants benefit from consuming these amino acids in appropriate proportions.^[32]^ Breastfeeding mothers can supplement their nutrition by obtaining sufficient EAAs and, in turn, meet their infants’ nutritional needs. However, except for 18AA-VII and 18AA-IX, for which the drug inserts stated that they may be used if the advantages of treatment and breastfeeding nutrition are greater than the risks, the drug inserts for the remaining medications were unclear. As a result, all 9 drugs were given a score of 0.5 for this criterion.

##### 3.3.2.5. Liver function abnormality

Compound amino acid injection can effectively improve liver function and provide effective and efficient nutritional support in patients with hepatic insufficiency.^[33,34]^ However, Chen Qin^[35]^ observed that 18AA-II caused liver function abnormality when treating a patient with a falling coma, referring to the instruction manual of 18AA-II suggesting caution for patients with hepatic and renal insufficiency, so it scored 2 points; the remaining 8 drugs are prohibited for patients with hepatic coma or tendency to hepatic coma, so they all scored 2 points.

##### 3.3.2.6. Abnormal renal function

Supplement 18AA-IX can benefit patients suffering from hypoproteinaemia, low nutritional status, and those undergoing surgery who have acute or chronic renal insufficiency, scoring 3 points. However, the other 8 medicines should not be used in patients with severe renal failure and should be used with caution in patients with renal insufficiency, earning only 2 points.

#### 3.3.3. Drug interactions

The main components of all 9 18AA compound amino acid injections are amino acids, which, as a nutritional support drug, are not dependent on hepatic metabolic enzyme systems primarily through direct participation in protein synthesis in vivo, and therefore do not induce or inhibit typical metabolic enzyme systems (e.g., CYP450), nor act as substrates for these enzymes. When combined with electrolyte solutions, antibiotics (e.g., aminoglycosides and tetracyclines), and other nutritional solutions (e.g., fat emulsions, dextrose solutions), they may affect electrolyte homeostasis, antibiotic efficacy, or the stability of the mixed solution,^[36]^ but most of these interactions are mild to moderate and usually do not require dosage adjustments, although indicators need to be monitored closely. Therefore, all 9 drugs in this item were scored as 3 points.

#### 3.3.4. Other attributes

18AA and its analogs meet the 3 points that all adverse reactions are reversible, no teratogenicity or carcinogenicity, and no special dosing warnings, thus scoring 3 points. The safety score results are shown in Table 6.

**Table 6:** Safety score results.

Safety (25 Points)	Grade	18AA	18AA-I	18AA-II	18AA-III	18AA-IV	18AA-V	18AA-V-SF	18AA-VII	18AA-IX
*Moderate adverse reactions (3 points*)										
Incidence < 1 %	3	3	3	3	3	3	3	3	3	3
Incidence 1%–<10%	2									
Incidence ≥ 10%	1									
ADR occurrence data not provided	0									
*Serious adverse reactions (5 points*)										
Incidence < 0.01%	5							5		
Incidence 0.01%–<0.1%	4	4	4	4	4	4	4		4	4
Incidence 0.1%–<1%	3									
Incidence 1%–<10%	2									
Incidence ≥ 10%	1									
ADR occurrence data not provided	0									
*Special populations (multiple choice, 11 points*)										
Available for children (2 points for all, 1.9 points for 3 mo and older, 1.8 points for 6 mo and older, 1.7 points for 9 mo and older, 1.6 points for 1 yr and older, 1.5 points for 2 yr and older, 1.4 points for 3 yr and older, 1.3 points for 4 yr and older, 1.2 points for 5 yr and older, 1.1 points for 6 yr and older, 1.0 points for 7 yr and older, 0.9 points for 8 yr and older, 0.8 points for 9 yr and older, 0.7 points for 10 yr and older, 0.6 points for 11 yr and older, 0.5 points for 12 yr and older).	2	2	2	2	0	0	2	2	1.9	1.9
Available for the elderly (1 point for available, 0.5 point for caution).	1	1	1	1	1	1	1	1	1	1
Available for women during pregnancy (1 point for early pregnancy, 0.8 points for midpregnancy, 0.5 points for late pregnancy).	1	0.5	0.5	0.5	0.5	0.5	0.5	0.5	0.5	0.5
Available for lactating women (1 point for availability, 0.5 points for caution).	1	0.5	0.5	0.5	0.5	0.5	0.5	0.5	0.5	0.5
Abnormal liver function available (3 points for severe available, 2 points for moderate available, 1 point for mild available).	3	2	2	2	2	2	2	2	2	2
Abnormal kidney function available (3 points for severe, 2 points for moderate, 1 point for mild).	3	2	2	2	2	2	2	2	2	3
*Adverse reactions due to drug interactions (3 points*)										
No dosage adjustment required	3	3	3	3	3	3	3	3	3	3
Dose adjustment required	2									
Prohibition of use during the same period of time	1									
*Other (multiple choice, 3 points*)										
Adverse effects are reversible	1	1	1	1	1	1	1	1	1	1
No teratogenicity or carcinogenicity	1	1	1	1	1	1	1	1	1	1
No special medication warnings	1	1	1	1	1	1	1	1	1	1
The results of safety	25	21	21	21	19	19	21	22	20.9	21.9

### 3.4. The economy

The economic study primarily compared the difference in average daily treatment costs between the selected drugs, drugs with the same generic name, and substitutable drugs for the primary indications. The dosage was calculated by the provided instructions, and except for 18AA-V-SF, the dosage for the remaining 8 drugs remained within a specific range. As a result, the average daily treatment costs were calculated by obtaining the average. The drug prices were obtained from various sources, including the latest 1-year publications (August 2022–2023) on drug prices in the Pharmaceutical Intelligence Network database, the Sunshine Procurement Platform, enterprise websites, the National Healthcare Security Bureau, and regional drug trading centers. The mean daily treatment costs (as presented in Table 7) and economic evaluations regarding the 9 drugs are detailed as follows: 18AA (Guangzhou Green Cross Pharmaceutical Co., Ltd., Guangzhou, China) exhibits the least expensive daily treatment cost among the 9 drugs; as such, it is used as the benchmark for evaluating the equivalent indications. The economy score results are shown in Table 8.

**Table 7:** Estimated average daily cost of treatment for 9 drugs.

Product name	Specification packaging	Dosage	Unit price (¥)	Average daily cost of treatment (¥)	Note
*Compound Amino Acid Injection (18AA*)	*250 mL*	*250–500/times/d*	*2.87*	*4.31*	*Guangzhou Green Cross Pharmaceutical Co.* *
Compound Amino Acid Injection (18AA-I)	250 mL	250–750/times/d	11.13	22.26	Cisen Pharmaceutical Co., Ltd.
Compound Amino Acid Injection (18AA-I)	250 mL	250–750/times/d	9.79	19.58	Guangdong Litai Pharmaceutical Co.*
Compound Amino Acid Injection (18AA-II)	250 mL	500–2000/times/d	13.57	67.85	Guangdong Litai Pharmaceutical Co.
Compound Amino Acid Injection (18AA-II)	250 mL	500–2000/times/d	7.57	37.85	Fuzhou Fu Yao Pharmaceutical Co., Ltd.*
Compound Amino Acid Injection (18AA-III)	250 mL	250–750/times/d	66.40	132.80	Guangdong Litai Pharmaceutical Co.*
Compound Amino Acid Injection (18AA-IV)	250 mL	500–1000/times/d	34.99	104.97	Guangdong Litai Pharmaceutical Co.*
Compound Amino Acid Injection (18AA-V)	250 mL	500–1500/times/d	10.25	41.00	Fuzhou Fu Yao Pharmaceutical Co., Ltd.*
Compound Amino Acid Injection (18AA-V)	250 mL	500–1500/times/d	6.05	24.20	Guangdong Litai Pharmaceutical Co.*
Compound Amino Acid Injection (18AA-V-SF)	250 mL	500/session/d	24.15	48.30	Hubei Yidian Pharmaceutical Co.*
Compound Amino Acid Injection (18AA-VII)	200 mL	200–800/times/d	31.23	78.08	Liaoning Haisco Pharmaceutical Co., Ltd.
Compound Amino Acid Injection (18AA-VII)	200 mL	200–800/times/d	4.34	77.45	Anhui Fubon Pharmaceutical Co.*
Compound amino acid injection (18AA-IX)	200 mL	200–400/times/d	89.00	133.50	Liaoning Haisco Pharmaceutical Co., Ltd.

Row in italics indicate the lowest cost of treatment per day for the same indication.

* Lowest cost of treatment per day for the same generic name.

**Table 8:** Economy score results.

Economy (10 points)	Grade	18AA	18AA-I	18AA-II	18AA-III	18AA-IV	18AA-V	18AA-V-SF	18AA-VII	18AA-IX
*Economy of the primary indication (10 points*)										
Evaluation method: 10 points for the drug with the lowest average daily cost of treatment, evaluation drug score = (lowest average daily cost of treatment/average daily cost of treatment of the evaluated drug) × 10.	10	10	3.99	2.56	3.23	3.29	2.75	3.62	3.37	3.23
The results of economy	10	10	3.99	2.56	3.23	3.29	2.75	3.62	3.37	3.23

### 3.5. Other attributes

Information has been gathered from various sources, including the 2018 edition^[37]^ of the National Basic Drug Catalog, the National Drug Catalog for Basic Medical Insurance, Workers’ Compensation Insurance and Maternity Insurance,^[38]^ the National Centralized Purchasing Drug Catalog, drug packaging, and instruction manuals, the China Listed Drugs Catalog Collection, and public announcements on the government websites of the NMPA and the Drug Evaluation Centre of the State Drug Administration. The Pharmaceutical Executive’s publication outlining the world’s top 50 pharmaceutical companies in terms of sales volume and the Ministry of Industry and Information Technology of the People’s Republic of China (MIIT)’s list of the 100 strongest pharmaceutical industries^[39]^ are both considered. Information on drug review approvals and drug listings can be obtained from official national websites such as the NMPA and the FDA.

18AA, 18AA-I, 18AA-II, 18AA-III, and 18AA-V are categorized as NHI Category A without payment restrictions and score 3 points. 18AA-VII is categorized as NHI Category B without payment restrictions and scores 2 points. The 18AA-V-SF is categorized as NHI Category B with payment restrictions and scores 1 point. The drugs 18AA-IV and 18AA-IX are not listed in the NHI catalog, scoring 5 points. On the other hand, 18AA is part of the National essential drugs (NED) and does not require a delta symbol, scoring 1 point. Additionally, none of the other drugs are listed in the NED catalog, scoring 3 points. The drugs selected were not included in the national centralized purchasing of selected drugs; therefore, this item was not scored. Through consistency of evaluation, 18AA-VII and 18AA-IX score 0.5 points, while none of the other drugs are scored. Cisen Technology Group Ltd., Jining, China is ranked 76th in the top 100 MIIT pharmaceutical industry list for 2021, scoring 0.4 points. The remaining drug manufacturers were not included in the MIIT top 100 pharmaceutical industry list or the top 50 pharmaceutical companies in the world in terms of sales volume, hence scoring zero points. All the drugs in the selection process are marketed and sold only domestically, and therefore, no points are awarded for these criteria. The other attributes’ score results are shown in Table 9.

**Table 9:** Other attributes score results.

Other attributes (10 points)	Grade		18AA	18AA-I		18AA-II	18AA-III		18AA-IV	18AA-V		18AA-V-SF	18AA-VII	18AA-IX
*NHI (3 points*)														
NHI Category A, no payment restrictions.		3	3		3	3		3			3			
NHI Category A has payment restrictions		2.5												
NHI Category B, no payment restrictions.		2											2	
NHI Category B, with payment restrictions.		1.5										1.5		
Not on the NHI catalogue		1							1					1
*NED (3 points*)														
NED, no requirement.		3	3											
NED, Requirements		2												
Not on the NED List		1			1	1		1	1		1	1	1	1
*National centralized procurement of medicines (1 point*)														
Selected medicines for centralized national procurement		1	0		0	0		0	0		0	0	0	0
*Original research/reference/consistency evaluation (1 point*)														
Drug of origin/reference		1												
Passing the consistency evaluation of generic drugs		0.5	0		0	0		0	0		0	0	0.5	0.5
*Status of production enterprises (1 point*)														
World’s top 50 pharmaceutical companies in terms of sales volume/MIIT’s top 100 companies in the pharmaceutical industry (Top 50 pharmaceutical companies in world sales 1–10 1, 11–20 0.8, 21–30 0.6, 31–40 0.4, 41–50 0.2; MIIT Pharmaceutical Industry Top 100 list) (enterprises 1–20 1, 21–40 0.8, 41–60 0.6, 61–80 0.4, 81–100 0.2)		1	0		0.4	0		0	0		0	0	0	0
*Global usage (1 point*)														
Available in China, USA, Europe, Japan		1												
Domestic and international sales		0.5	0		0	0		0	0		0	0	0	0
Other attribute scores	10		6	4.4		4	4		2	4		2.5	3.5	2.5

MIIT = Ministry of Industry and Information Technology, NED = National Essential Drug, NHI = National Health Insurance.

### 3.6. Final scoring results for the 5 dimensions of the nine 18AA compound amino acid injection

Table 10 displays the final scores for the nine 18AA compound amino acid injections evaluated. The top 3 medications were obtained by 18 AA, 18 AA-VII, and 18 AA-IX.

**Table 10:** Final score results.

Evaluation dimension	18AA	18AA-I	18AA-II	18AA-III	18AA-IV	18AA-V	18AA-V-SF	18AA-VII	18AA-IX
Pharmacological properties	20.5	20	20	19	19	19	19	21	21
Efficacy	14	14	14	16	12	12	14	18	18
Safety	21	21	21	19	19	21	22	20.9	21.9
Economy	10	3.99	2.56	3.23	3.29	2.75	3.62	3.37	3.23
Other attributes	6	4.4	4	4	2	4	2.5	3.5	3.5
Total	71.5	63.39	61.56	61.23	55.29	58.75	61.12	66.77	66.63

## 4. Discussion

### 4.1. Analysis of results

The results of the assessment indicated that 18 AA received the highest score. This drug has been on the market for an extended period and is the only evaluated medication featured in the NED Catalog and the NHI Category A. Additionally, it boasts several advantages, such as its significant price advantage, high applicability to special populations, and a composite score of 71.5. Therefore, it is highly recommended that 18 AA be included in the drug use catalog of healthcare institutions. Eighteen AA-VII, 18 AA-IX, 18 AA I, 18 AA-II, 18 AA-III, and 18 AA-V-SF are available for clinical use, with a comprehensive score ranging from 60 to 70. Specifically, 18 AA-VII and 18 AA-IX have been developed to address the metabolic characteristics of certain diseases and can be used for targeted amino acid supplementation. The other 2 drugs scored <60 and are therefore not recommended for inclusion in the Drug Use Catalog for medical institutions.

The evaluation outcomes aim to offer a scientific foundation for hospital personnel to choose 18AA compound amino acid injection based on rational decision-making. Additionally, the evaluation outcomes can foster the optimization of drug variety composition in healthcare institutions and the hospital drug catalog. Concurrently, the evaluation results are of great significance to clinicians since they provide an evidence-based basis.

### 4.2. Comparative analysis with other studies

Our study evaluated 9 compounded amino acid formulations for injection, which is consistent with the general trend of the results of another study on the evaluation of balanced compounded amino acids.^[40]^ The other study compared 6 balanced compounded amino acid injections on only 3 dimensions of efficacy, safety, and economics, and although there were differences in the evaluation methodology and scope of inclusion, both studies emphasized the importance of sulfite content for safety, concluded that 18AA offered the best economic value, and both studies agreed that 18AA-V and 18AA-IV performed in all 3 dimensions were poorer. Notably, it is noteworthy that although our study found that 18AA-IX and 18AA-VII scored the highest on pharmacological properties and efficacy and that 18AA-IX and 18AA-V-SF excelled on safety, these top-performing compounded amino acid injections were not included in the comparative study, and the results of the remaining compounded amino acid injection evaluations in the 2 studies showed a consistent overall trend, which demonstrates the reliability of our findings. Our study provides a broader perspective by evaluating a wider range of formulations and using a comprehensive set of criteria. These differences highlight the importance of considering a variety of evaluation methods and dimensions to provide balanced, practical recommendations for clinical use.

### 4.3. Limitations

#### 4.3.1. Limitations of data sources

The data source of this study is in Chinese, and some of the information may lack completeness and accuracy. The evaluation system is established for China’s national conditions and may provide drug selection suggestions and methodological references for China or other low- and middle-income countries or countries with lower levels of medical care, as well as medical institutions that cannot carry out drug evaluation on their own. However, it may not be generalizable to the medical practice of developed countries or medical institutions with a sound drug evaluation system.

#### 4.3.2. Limitations of the research method

This study utilized a quantitative scoring method to evaluate the effectiveness of compound amino acid injection. However, subjective factors may have impacted the evaluation results. For instance, relevant RCTs and META analyses of compound amino acid injection, which could contribute to the evaluation of drug efficacy, have scarcely been studied. Additionally, the primary and secondary efficacy endpoints reported in many studies are not uniform. In addition, this study did not conduct any clinical trials to directly evaluate the drug’s clinical efficacy and safety. Furthermore, there were inconsistencies in the grading of specific populations. For instance, pregnant women were graded based on the availability of care during early, middle, and late pregnancy, without considering the difference between availability and appropriate use.

#### 4.3.3. Timeliness of data

The final scores obtained from this selection guide are time-sensitive. For instance, drug inserts are less detailed about adverse reactions, and national ADR data are updated accordingly; hence, they may not be reflective of the most recent situation. As evidence-based guidance, such as expert consensus or guidelines, and ADR reports are modified, the final score utilizing the selection guide may also be altered. Moreover, as a result of varying policies and hospital management models across different regions of China, combined with fluctuating prices resulting from updates to the winning bids of the national or provincial collections, medication prices remain dynamic. Therefore, when prescribing drugs, it is crucial to monitor these price changes and discuss and reevaluate medication use when necessary, while simultaneously considering the unique needs and factors influencing each healthcare organization and region.

### 4.4. Summary and outlook

Overall, there are still limited studies on multidimensional comprehensive evaluation of different compounded amino acid injections. Our study not only evaluated 9 different compounded amino acid injections, but also provided a comprehensive set of evaluation criteria, which fills the research gap regarding the comprehensive evaluation of compounded amino acid injections and provides a basis for quantitatively evaluating compounded amino acid injections used for hospital selection. This study can provide a strong evidence-based medical reference and support when healthcare organizations need to make quick decisions. Future research could investigate additional attributes of compounded amino acid injections, such as drug stability, solubility, and compatibility. Furthermore, a broader range of compounded amino acid injections should be evaluated to expand the scope of the study and provide more alternative options. The formulation and usage of compounded amino acid injections can also be optimized based on clinical practice and patient needs to improve their clinical efficacy and safety.

## 5. Conclusion

When a healthcare organization introduces 18AA compounded amino acid injections to their hospital, they can refer to the assessment results and use the top 3 recommended medications: 18 AA, 18 AA-VII, and 18 AA-IX. Meanwhile, 18AA, which has the highest evaluation score and is >70, should be highly recommended as a new drug introduction and prioritized during drug adjustment and retention. However, this study is based on China’s national conditions, and the results are mainly applicable to Chinese medical practice, with limited international applicability. In the future, more extensive research is needed to verify the applicability of these conclusions in different healthcare systems.

## Acknowledgments

We would like to thank all of the authors who participated in the present study.

## Author contributions

**Conceptualization:** Jiabao Li, Siyong Huang, Jisheng Chen.

**Data curation:** Siyong Huang.

**Formal analysis:** Jiabao Li.

**Funding acquisition:** Jisheng Chen.

**Investigation:** Xiao Hu, Siyong Huang.

**Methodology:** Xiao Hu, Siyong Huang.

**Project administration:** Xiao Hu, Siyong Huang.

**Resources:** Jiabao Li, Xiao Hu, Siyong Huang.

**Software:** Jiabao Li.

**Supervision:** Jisheng Chen.

**Validation:** Jiabao Li, Jisheng Chen.

**Visualization:** Jiabao Li.

**Writing – original draft:** Jiabao Li.

**Writing – review & editing:** Jiabao Li, Jisheng Chen.
